# Fluralaner (Bravecto®) induces long-term mortality of *Lutzomyia longipalpis* after a blood meal in treated dogs

**DOI:** 10.1186/s13071-020-04489-1

**Published:** 2020-12-04

**Authors:** Tamyres Bernadete Dantas Queiroga, Henrique Rafael Pontes Ferreira, Wilo Victor dos Santos, Ana Beatriz Lourenço de Assis, Vicente Toscano de Araújo Neto, Antônia Cláudia Jácome da Câmara, João Ciro Fagundes Neto, Romeika Karla dos Reis, Manuela Sales Lima Nascimento, Renata Antonaci Gama, Paulo Marcos Matta Guedes

**Affiliations:** 1 Graduate Program in Pharmaceutical Sciences, Federal University of Rio Grande do Norte, Natal, Rio Grande do Norte Brazil; 2 Graduate Program in Parasitary Biology, Federal University of Rio Grande do Norte, Natal, Rio Grande do Norte Brazil; 3 Department of Clinical and Toxicological Analyses, Federal University of Rio Grande do Norte, Natal, Rio Grande do Norte Brazil; 4 Zoonoses Control Center, Natal, Rio Grande do Norte Brazil; 5 Canis and Catus Veterinary Clinic, Natal, Rio Grande do Norte Brazil; 6 Department of Microbiology and Parasitology, Federal University of Rio Grande do Norte, Natal, Rio Grande do Norte Brazil

**Keywords:** Visceral leishmaniasis, Fluralaner, Bravecto®, Systemic insecticide, *Lutzomyia longipalpis*, Sand fly, Dog

## Abstract

**Background:**

*Leishmania infantum* is the etiological agent of visceral leishmaniasis (VL) in the New World, where the sand fly *Lutzomyia longipalpis* and domestic dogs are considered the main vector and host reservoirs, respectively. Systemic insecticides have been studied as an alternative to control vector-borne diseases, including VL. Fluralaner, an isoxazoline class compound, is a systemic insecticide used in dogs, with proven efficiency against different species of phlebotomine sand flies. However, to date no studies have demonstrated the efficacy of fluralaner on *Lu. longipalpis*. The aim of this study was to evaluate the insecticidal effect of fluralaner (Bravecto®) on the sand fly *Lu. longipalpis* after blood meal in treated dogs.

**Methods:**

Healthy mongrel dogs (*n* = 8) were recruited from the Zoonoses Control Center in the city of Natal, Rio Grande do Norte, Brazil, and randomized into two groups: fluralaner treated (*n* = 4) and non-treated control (*n* = 4). Colony-reared female specimens of *Lu. longipalpis* (*n* = 20) were allowed to feed on all dogs for 40 min before treatment (for fluralaner-treated dogs), at day 1 after treatment and then monthly until 1 year post-treatment.

**Results:**

In the treatment group, there was 100% mortality of *Lu. longipalpis* for up to 5 months after treatment initiation, decreasing to 72.5% at 6 months post-treatment initiation. The efficacy of fluralaner ranged from 100% at day 1 (*P* = 0.0002) to 68% ( *P* = 0.0015) at 6 months, decreasing to 1.4% at 1 year post-treatment. Sand fly mortality carried out blood meal in non-treated control dogs remained constant at ≤ 15%.

**Conclusions:**

Taken together, our results suggest that fluralaner may be used as a control strategy for VL in dogs in VL endemic areas. 
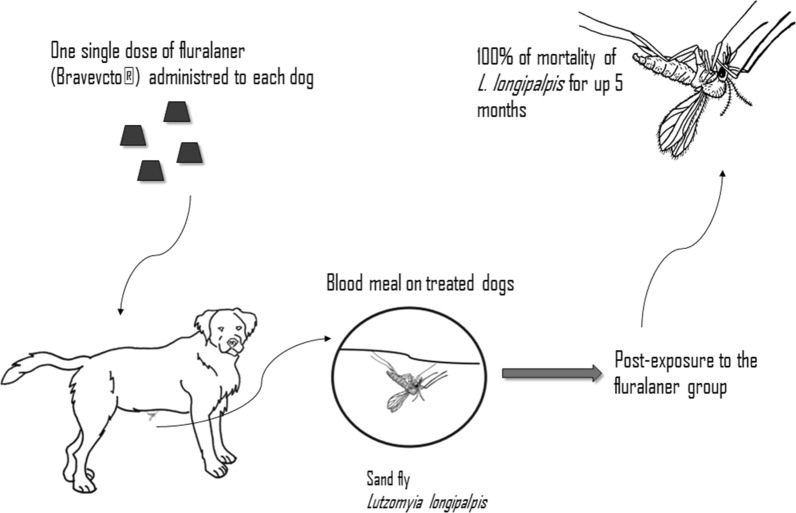

## Background

Visceral leishmaniasis (VL) is caused by the protozoan parasite *Leishmania infantum* and is considered to be a public health concern due to its wide geographic distribution, high number of estimated cases annually (50,000–90,000) and high mortality rate, mainly in malnourished children, old-aged individuals and patients with *Leishmania*–human immunodeficiency virus coinfection. In the Americas, VL is endemic in 12 countries, with approximately 96% of new cases reported in Brazil [1–3]. Dogs are the main domestic reservoir and play an important role in maintaining the disease cycle due to the high prevalence of *L. infantum* infection in dogs, the presence of amastigote forms in their skin and their proximity to humans [4–7]. For this reason, dogs are considered to be strategic targets for disease control.

The main *L. infantum* transmission route is through the bite of the blood-feeding female phlebotomine sand fly (Diptera, Psychodidae, Phlebotominae) belonging to the genus *Phlebotomus* in the Old World and *Lutzomyia* in the New World [8]. *Lutzomyia longipalpis* is the main vector of *L. infantum* in the Americas [7]. This sand fly species is widely distributed throughout most Brazilian states, and has shown to be highly adaptable to the urban environment [9–12]. The control actions currently used to target the vector and its main reservoir are controversial and appear to have a limited impact on the incidence of human and canine VL cases due to several limitations, including inefficiency of diagnostic methods, absence of a treatment that eliminates the parasite and/or lack of an effective vaccine to prevent infection in humans and dogs [13, 14].

Previous studies have explored the use of systemic insecticides as a control measure. Recently, some studies have focused on a new class of drugs, isoxazolines (fluralaner, afoxolaner, saxolaner), which are licensed for veterinary use to protect against fleas, ticks and mites through its inhibition of the arthropod nervous system [15–17]. Among this class of drugs, the fluralaner compound provides the most prolonged protection, approximately 12 weeks [18–20]. Fluralaner acts as an antagonist of the gamma-aminobutyric acid (GABA)-gated chloride ion (Cl^−^) channels, preventing the entry of Cl^−^ into the postsynaptic neuron, which in turn leads to hyperexcitability of the insect central nervous system [15]; it has a very long half-life* in vivo* [21]. Fluralaner has been shown to be safe in dogs [22] and has a significant selectivity for insects over mammalian receptors [23]. Experimental studies with fluralaner have shown insecticide activity within short periods of time against *Triatoma infestans*, the vector of *Trypanosoma cruzi* (the etiologic agent of Chagas disease [24]) and against some species of phlebotome sand flies, such as *Phlebotomus papatasi* [25, 26] and *P. perniciosus* [27]. However, to date, there has been no study demonstrating fluralaner action on *Lu. longipalpis*, the main vector of VL in the New World. In this study, we evaluated the systemic insecticidal activity of a single oral dose of fluralaner against *Lu. longipalpis* after blood feeding on dogs, based on monthly assays of *Lu. longipalpis* activity during 1 year.

## Methods

### Dog recruitment and maintenance

Dogs were obtained from the Zoonoses Control Center (ZCC) in the city of Natal, Rio Grande do Norte (Brazil). With permission of the ZCC, eight healthy mongrel dogs (*n* = 8; 1:1 ratio of male and female), weighing ≥ 20 kg were recruited and maintained in individual kennels. They were provided with water *ad libitum* and food corresponding to 5% of their body weight and routinely observed. No insecticide treatment was used in the kennels or on the dogs during the assays, and the animals were bathed with neutral shampoo 2 weeks before each assay.

### Sand fly colony maintenance

A sand fly colony was established in the Laboratory of Insects and Vectors of the Federal University of Rio Grande do Norte (Brazil), starting with eggs of *Lu. longipalpis* (donated by the Laboratory of Physiology of Hematophagous Insects of the Federal University of Minas Gerais, Brazil). The colony was maintained under standard conditions (75–80% relative humidity, 26–27 ℃, 12:12 light:dark cycle). A homogeneous fermented mixture consisting of crushed mouse food, dried rabbit feces and soil was used for larval feeding [28].

### Study design

The dogs were randomized into two groups: a fluralaner-treated (*n* = 4) group and an untreated control group (*n* = 4), with each group comprising two male and two female dogs. Randomization was by tossing a coin in the presence of an observer. The fluralaner-treated group received a single oral dose of fluralaner (Bravecto®; Merck Animal Health, Merck Sharp & Dohme, Kenilworth, NY, USA) on the same day as randomization, following the manufacturers’ recommendations. The sand fly feeding assays were performed on both groups 1 h prior to fluralaner treatment and on days 1, 30, 60, 90, 120, 150, 180, 210, 240, 270, 300, 330 and 360 (i.e. monthy assessments) to evaluate mortality.

**Figure 1:**
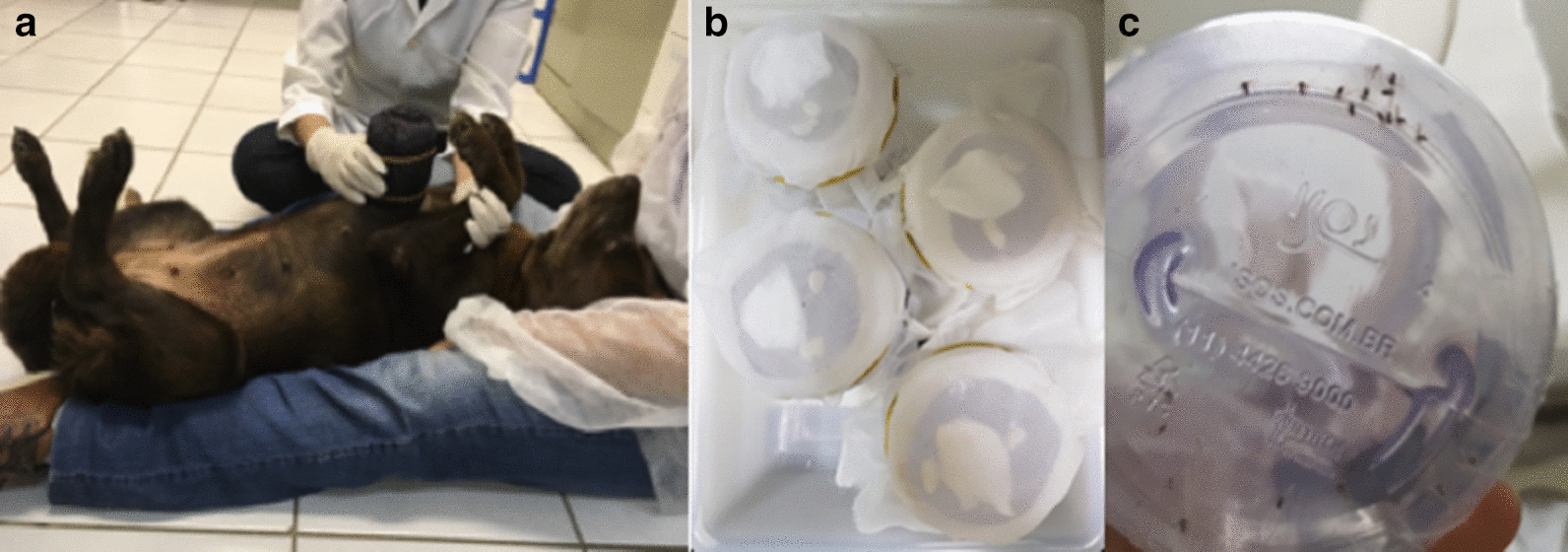
Entomological bioassay. *Lutzomyia longipalpis* specimens blood-fed directly on dogs treated with fluralaner.** a** A cylindrical plastic pot covered with a net, containing 20 female/5–10 male specimens of *Lu. longipalpis*, was positioned onto the ventral region of each dog for 40 min.** b** Insects were fed with cotton soaked with a glucose-saturated solution, maintained and monitored in an insectary under standard conditions and assays at 24, 48, 72, 96 and 120 h post-exposure to determine mortality rate.** c** Dead specimens of *Lu. longipalpis* after the observational period.

Female sand flies (5–6 days old) of *Lu. longipalpis*, without blood- or sugar-feeding (12 h before the experiment), were used. The dogs were gently restrained by trained veterinarians and positioned upright or lying down for more comfortable position. This was followed by the placement of a cylindrical plastic pot fitted with a tight lid that was perforated and covered with a net onto the ventral region of each dog, with each pot containing 20 female/5–10 male specimens of *Lu. longipalpis*, which were allowed to feed for 40 min (Fig. 1a). After the assays, sand flies were maintained in an insectary under standard conditions (relative humidity 75–80 %, 26–27 °C and 12:12 light:dark cycle), and the insects were fed with cotton soaked in a glucose-saturated solution (Fig. 1b). All female specimens were monitored at 24, 48, 72, 96 and 120 h post-blood meal to record the number of dead females, determined by the lack of movement (Fig. 1c).

### Statistical analysis

The efficacy of fluralaner for killing sand flies was assessed using Abbott’s formula [29]. Efficacy was measured by comparing the number of dead female insects after a blood meal from the treated and untreated group using the following formula:

$${\text{Efficacy }}\left( \% \right) \, = \, \left( {1 - \frac{{n{\text{ in T after treatment}}}}{{n{\text{ in Co after treatment}}}}} \right) \, \times { 1}00$$

where *n* is the sand fly population, T is treated (dogs) and Co is the control (dogs). Differences between the groups were determined using Fisher’s exact test on each experimental test day [25]. Sand fly mortality tests were two-tailed and differences between groups were considered to be significant when *P* ≤ 0.05. Survival analyses were conducted using the Kaplan Meier method on each assay day until 120 h post-feeding. The analyses were performed using BioEstat 5.0 software (Brazil).

## Results

### No side effects were observed in dogs treated with fluralaner

The assays were conducted between November 2018 and November 2019. Recruited dogs weighed between 20 and 40 kg and aged between 4 and 10 years. There were no significant differences in the weight or ages of dogs between the untreated control and fluralaner-treated dogs. No side effects among fluralaner-treated dogs were observed during the study.

### Fluralaner (Bravecto®) induces 100% mortality of *Lu. longipalpis* up to 5 months after treatment in dogs

**Fig. 2:**
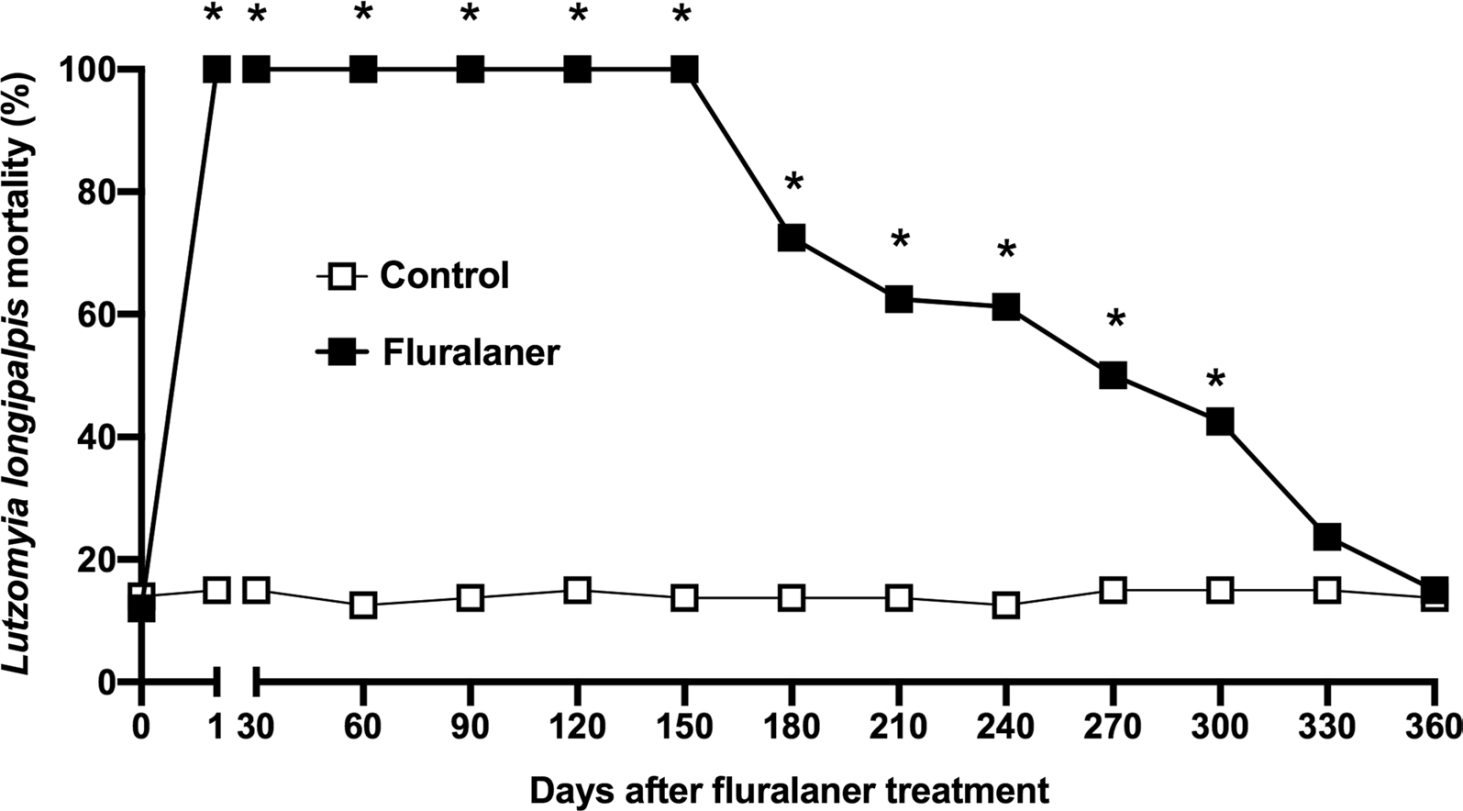
*Lutzomyia longipalpis* mortality (%) after blood meal on fluralaner-treated dogs and untreated control dogs. After fluralaner-treated dogs had received a single oral dose of fluralaner (Bravecto®), *Lu. longipalpis* mortality (*n* = 20) was assessed on days 1, 30, 60, 90, 120, 150, 180, 210, 240, 270, 300, 330 and 360 post-treatment. The data are representative of two-tailed tests and Fisher’s exact test was used. Asterisk (*) indicates a significant difference between groups at *P* < 0.05

All female *Lu. longipalpis* specimens used in the assays were engorged, with 100% feeding success. Following the administration of a single oral dose of fluralaner to dogs, during the 1-year follow-up study we recorded 100% mortality of female *Lu. longipalpis* specimens up to 150 days after treatment (Fig. 2). After the fifth month, mortality levels started to decline with increasing time from treatment, to 72.5% on post-treatment day 180, 62.5% on post-treatment day 210, 61.2% on post-treatment day 240, 50% on post-treatment day 270, 42.5% on post-treatment day 300, 23.7% on post-treatment day 330 and 15% on post-treatment day 360. The mortality of the sand flies after the blood meal in treated dogs was higher than that in the control dogs until post-treatment day 300 (Fig. 2). However, mortality was similar between groups at post-treatment days 330 and 360 (Fig. 2). The mortality of sand flies blood-fed on control dogs was always ≤ 15% (range 12.5–15%) up to 120 h after the feeding (Fig. 2).

**Fig. 3:**
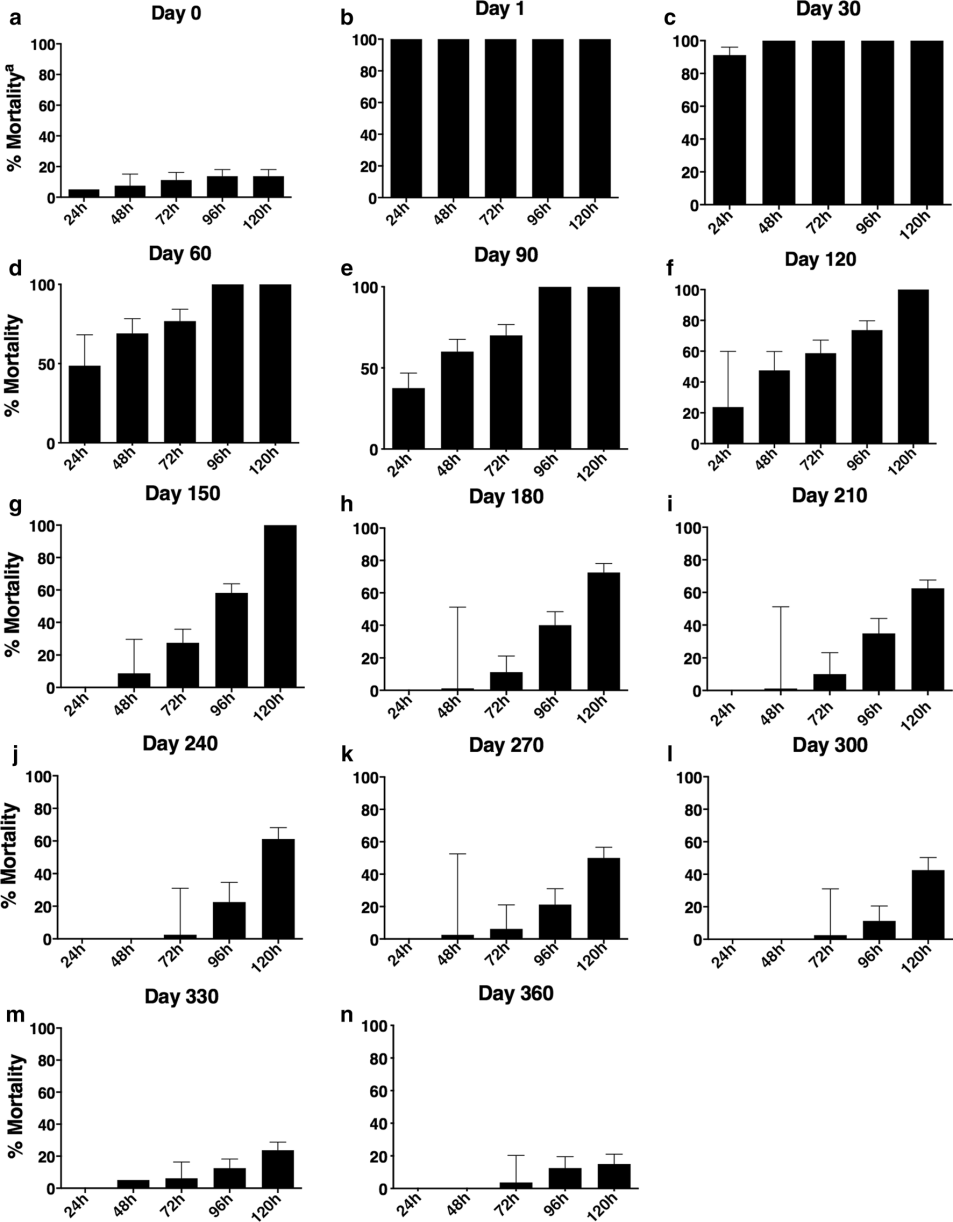
*Lutzomyia longipalpis* mortality up to 120 h after blood meal in dogs treated with fluralaner (Bravecto®). Proportion (%) of *Lu. longipalpis* (*n* = 20) specimens that died within 24, 48, 72, 96 and 120 h after blood meal on mongrel dogs treated (*n* = 4) and not treated (*n* 4/control) with fluralaner at post-treatment days 1, 30, 60, 90, 120, 150, 180, 210, 240, 270, 300, 330 and 360

Analyses of *Lu. longipalpis* mortality was performed in each experiment until 120 h after blood-feeding on dogs. On the first day post-treatment, 100% of the sand flies had died by 24 h after the blood meal on the treated dogs (Fig. 3). After post-treatment day 1, the time to reach 100% mortality gradually increased, to 48 h on post-treatment day 30, 72 h on post-treatment day 60, 96 h on post-treatment day 90, 120 h on post-treatment day 120 and 120 h on post-treatment day 150. Fluralaner provided sustained mortality (100%) for up to 5 months according to the 120 h post-test (Fig. 3). Mortality results observed after 120 h were 72.5% on post-treatment day 180, 62.5% on post-treatment day 210, 61.2% on post-treatment day 240, 50% on post-treatment day 270, 42.5% on post-treatment day 300, 23.7% on post-treatment day 330, and 15% on post-treatment day 360 (Fig. 3).

### Fluralaner (Bravecto®) has insecticidal efficacy against *Lu. longipalpis* for up to 9 months after treatment in dogs

The fluraner-treated dogs had a significantly higher insecticidal efficacy between post-treatment day 1 (*P* = 0.0002) and day 150 (*P* < 0.0001), with insecticidal efficacy maintained at 100%, than during the subsequent follow-up, with efficacy decreasing to 68.1% on post-treatment day 180 (*P* *=* 0.0015), 56.5% on post-treatment day 210 (*P* = 0.0049), 55.7% on post-treatment day 240 (*P* = 0.0022) and 41.2% on post-treatment day 270 (*P* = 0.0171) (Table 1). No significant differences in insecticidal efficacy were observed in the fluralaner-treated group on post-treatment days 300, 330 and 360 compared to the control group (Table 1). Kaplan–Meier analyses at 120 h post-feeding (95% confidence interval) per group and sampling day are reported in Table 1; the complete post-feeding data set (24, 48, 72, 96, 120 h) is provided in Additional file 1: Table S1. Taken together, these results show that the treatment of dogs with fluralaner is effective against *Lu. longipalpis* up to 270 days after treatment.

**Table 1:** Follow-up of *Lutzomyia longipalpis* mortality and insecticidal efficacy after blood meal on dogs for up to 12 months (360 days) after treatment with a single oral dose of fluralaner (Bravecto®)

Days after treatment	Control		Fluralaner		Efficacy (%)^c^	*P* value^d^
	Mortality (%)^a^	95% Confidence interval^b^	Mortality (%)^a^	95% Confidence interval^b^		
1	12 (15)	0.77–0.92	80 (100)	NA^e^	100	0.0002*
30	12 (15)	0.77–0.92	80 (100)	NA	100	0.0002*
60	10 (12.5)	0.80–0.94	80 (100)	NA	100	< 0.0001*
90	11 (13.75)	0.78–0.93	80 (100)	NA	100	< 0.0001*
120	12 (15)	0.77–0.92	80 (100)	NA	100	0.0002*
150	11 (13.75)	0.78–0.93	80 (100)	NA	100	< 0.0001*
180	11 (13.75)	0.77–0.92	58 (72.5)	0.17–0.37	68.1	0.0015*
210	11 (13.75)	0.78–0.93	50 (62.5)	0.26–0.48	56.5	0.0049*
240	10 (12.5)	0.80–0.94	49 (61.25)	0.28–0.49	55.7	0.0022*
270	12 (15)	0.77–0.92	40 (50)	0.39–0.60	41.2	0.0171*
300	12 (15)	0.77–0.92	34 (42.5)	0.46–0.68	32.3	0.0590
330	12 (15)	0.77–0.92	19 (23.75)	0.66–0.85	10.3	0.0301
360	11 (13.75)	0.78–0.93	12 (15)	0.77–0.92	1.4	1.0000

^a^Mortality indicates the number of sand flies dead at the specified time point. Presented as the number of dead sand flies with the percentage [(dead females after blood-feeding/total number of blood-fed females)  × 100] in parentheses

^b^95% Confidence interval is the interval of the probability of survival at the specified time point calculated using the Kaplan–Meier method

^c^Efficacy is the percentage of sand fly mortality after exposure to the insecticide using Abbott’s formula

^d^*P* value: Significance of difference in sand fly mortality between control group and fluralaner group using Fisher’s exact test. *Significant difference between treated dogs and control dogs at *P* ≤  0.05

^e^NA: Samples showed 100% death rate

## Discussion

In this study, we observed that a single oral dose of fluralaner induces *Lu. longipalpis* mortality, with insecticidal efficacy maintained up to 270 days after treatment in dogs. In the treated dogs, fluralaner treatment had an efficacy of 100% on female phlebotomine sand flies for the first 5 months post treatment, decreasing to 68.1% at 6 months. These results suggest that fluralaner may be a promising tool for VL control in dogs in endemic areas, possibly by administering the agent every 5 months.

There was a 100% mortality rate in *Lu. longipalpis* within 120 h post-blood meal on fluralaner-treated dogs for up to 150 days post-treatment. Susceptibility to fluralaner has been determined in other sand fly species, such as *Phlebotomus papatasi and P. perniciosus* [25, 27]. A survey by Gomez and co-workers noted mortality of 100, 96, 89 and 42% in *P. papatasi* (24 h post-blood meal directly on dogs) on post-treatment days 3, 31, 45 and 73, respectively [25]. We observed similar results in our study during the same period of time (24 h after blood meal) for *Lu. longipalpis*, with observed mortality of 100, 91.2, 48.7 and 37.5 on post-treatment days 1, 30, 60 and 90, respectively. Furthermore, a study with *P. perniciosus* showed that 96 h after blood meal, there was 100, 100 and 68.6% mortality on days 1, 28 and 84 post-fluralaner treatment, respectively [27]. In our study, 96 h after a blood meal, 100% mortality was recorded in *Lu. longipalpis* on days 1, 30 and 90 after fluralaner treatment, indicating that *Lu. longipalpis* is more susceptible to the insecticide effect of fluralaner than *P. perniciosus.* The insecticidal efficacy of fluralaner against *P. papatasi* was determined in a membrane-feeding study. Sand fly mortality 24 h post-feeding was higher in the group fed blood samples from fluralaner-treated dogs up to 30 days (60% mortality) post-treatment compared to the control [26].

In the present study, fluralaner was able to kill the female specimens of sand flies within 5 days after blood-feeding. Female sand flies take approximately 7 days to become infected after ingesting a blood meal, with development of the infective form in the foregut and possibly transmitting *L. infantum* to other hosts [30]. The use of fluralaner will not prevent an infected sand fly from transmitting the parasite during blood-feeding, but when used on a large scale it can become an alternative strategy to control *Lu. longipalpis* populations in risk areas in the long term because sand flies blood-feeding on fluralaner-treated dogs will die before becoming infectious, thereby preventing the transmission. In addition to furalaner’s action in adult sand flies, engorged sand fly females which did not die within the 120-h post-blood feeding period on treated dogs (> day 150 post-treatment) were able to lay eggs, although fewer eggs and larvae were observed. These observations suggest that fluralaner could act in preventing egg development and maturation (data not shown). However, further studies should be performed to elucidate this possible action.

We recorded the efficacy of fluralaner efficacy to kill *Lu. longipalpis* after blood-feeding on treated dogs for up to 270 days and observed 100% fluralaner efficacy against *Lu. longipalpis* 96 h after a blood meal. On the other hand, fluralaner efficacy against sand fly *P. perniciosus* was 100, 100 and 52.7 on days 1, 28 and 84 post-treatment, respectively [27]. The prolonged efficacy of fluralaner against *Lu. longipalpis*, when compared to other species of sand flies, may be associated with intra- and inter-specific variations in size or ingestion of the blood meal and the attractiveness of the animal under study [31, 32]. A study developed by William and co-workers showed that ticks collected from fluralaner-treated dogs weighed significantly less than ticks collected from the control dogs, leading to minimal blood absorption, which results in a sufficient exposure of ticks to the fluralaner and consequently high levels of mortality [33]. In the present study, we observed that all specimens showed high levels of mortality after feeding on fluralaner-treated dogs, regardless of the quantity of blood ingested by the *Lu. longipalpis* females. In addition, we performed a sand fly feeding assay, with 100% of feeding success, directly on the ventral region of dogs, where there is a greater concentration of fat and consequently a higher drug concentration than in other tissues [34]. In future studies, we intend to perform the feeding assays directly on the ear of the dogs and compare the results to assays on other body regions.

When evaluating systemic insecticides to be applied to dogs as a control measure for vector-borne diseases, some points should be considered. To achieve greater coverage in risk areas, insecticides should be easy to apply, present immediate and prolonged efficacy and require a low number of reapplications. When there is a good safety profile, side effects to the dog are avoided, increasing owner’s adherence [35]. In this context, fluralaner appears to be a promising compound for sand fly control, especially *Lu. longipalpis* populations. The results of our study show high levels of mortality, the longest reported residual activity against sand flies and high efficacy levels for up to 9 months. Another point to be mentioned is that only mongrel dogs were used in the study, presenting variable ages and weights, so the results may closely resemble the real-life situation.

## Conclusions

Taken together, our results show that fluralaner treatment in dogs has an insecticide efficacy against *Lu. longipalpis* and represents a possible public health intervention strategy for combating and reducing infected vectors in endemic areas of VL, thereby reducing canine and human cases of this disease.

## Supplementary information

**Additional file 1:** **Table S1.** *Lutzomyia longipalpis * mortality (%) after blood meal (determined up to 120 h after blood meal) on fluralaner (Bravecto®)-treated dogs for up to 12 months post-treatment.

## Data Availability

The datasets used and/or analyzed during the current study are available from the corresponding author on reasonable request.

## Abbreviations

NA: Not applicable
VL: Visceral leishmaniasis
ZCC: Zoonoses Control Center
