# Supplementary material for: Fluralaner (Bravecto®) induces long-term mortality of Lutzomyia longipalpis after a blood meal in treated dogs
Source: Parasit Vectors. 2020 Dec 4;13:609. doi: 10.1186/s13071-020-04489-1 (PMC7716428; doi:10.1186/s13071-020-04489-1)
Supplement: Supplementary file 1 — Additional file 1: Table S1. Lutzomyia longipalpis mortality (%) after blood meal (determined up to 120 h after blood meal) on fluralaner (Bravecto®)-treated dogs for up to 12 months post-treatment. [file 13071_2020_4489_MOESM1_ESM.docx]

**Table S1 –** **Follow-up *Lutzomyia longipalpis* mortality (%) after blood meal on dogs 12 months post-treatment with fluralaner (Bravecto).**

| Group |  | Days after treatment | | | | | | | | | | | | | |
| --- | --- | --- | --- | --- | --- | --- | --- | --- | --- | --- | --- | --- | --- | --- | --- |
|  |  | Day  0 | Day  1 | Day  30 | Day  60 | Day  90 | Day  120 | Day  150 | Day 180 | Day 210 | Day  240 | Day  270 | Day 300 | Day  330 | Day  360 |
| Control | Time | Deaths (%)  95% CI | | | | | | | | | | | | | |
|  | 24h | 0 (0)  0.99-1.00 | 4 (5)  0.90-0.99 | 6 (7.5)  0.86-0.98 | 5 (6.25)  0.88-0.99 | 3 (3.75)  0.92-1.00 | 2 (2.5)  0.94-1.00 | 3 (3.75)  0.92-1.00 | 4 (5)  0.90-0.99 | 4 (5)  0.90-0.99 | 5 (6.25)  0.88-0.99 | 6 (7.5)  0.86-0.98 | 4 (5)  0.90-0.99 | 4 (5)  0.90-0.99 | 4 (5)  0.90-0.99 |
|  | 48h | 3 (3.75)  0.92-1.00 | 0 (5)  0.90-0.99 | 3 (11.25)  0.81-0.95 | 2 (8.75)  0.85-0.97 | 2 (6.25)  0.88-0.99 | 2 (5)  0.90-0.99 | 2 (6.25)  0.88-0.99 | 2 (7.5)  0.86-0.98 | 1 (6.25)  0.88-0.99 | 2 (8.75)  0.85-0.97 | 2 (10)  0.83-0.96 | 0 (5)  0.90-0.99 | 4 (10)  0.83-0.96 | 1 (6.25)  0.88-0.99 |
|  | 72h | 4 (8.75)  0.85-0.97 | 4 (10)  0.83-0.96 | 0 (11.25)  0.81-0.95 | 1 (10)  0.83-0.96 | 3 (10)  0.83-0.96 | 3 (8.75)  0.85-0.97 | 3 (10)  0.83-0.96 | 3 (11.25)  0.81-0.95 | 3 (10)  0.83-0.96 | 1 (10)  0.83-0.96 | 1 (11.25)  0.81-0.95 | 4 (10)  0.83-0.96 | 0 (10)  0.83-0.96 | 3 (10)  0.83-0.96 |
|  | 96h | 3 (12.5)  0.80-0.94 | 2 (12.5)  0.80-0.94 | 0 (11.2)  0.81-0.95 | 2 (12.5)  0.80-0.94 | 2 (12.5)  0.80-0.94 | 4 (13.75)  0.78-0.93 | 2 (12.5)  0.80-0.94 | 3 (13.75)  0.77-0.92 | 2 (12.5)  0.80-0.94 | 2 (12.5)  0.80-0.94 | 0 (11.25)  0.81-0.95 | 2 (12.5)  0.80-0.94 | 2 (12.5)  0.80-0.94 | 2 (12.5)  0.80-0.94 |
|  | 120h | 2 (15)  0.77-0.92 | 2 (15)  0.77-0.92 | 3 (15)  0.77-0.92 | 0 (12.5)  0.80-0.94 | 1 (13.75)  0.78-0.93 | 1 (15)  0.77-0.92 | 1 (13.75)  0.78-0.93 | 0 (13.75)  0.77-0.92 | 1 (13.75)  0.78-0.93 | 0 (12.5)  0.80-0.94 | 3 (15)  0.77-0.92 | 2 (15)  0.77-0.92 | 2 (15)  0.77-0.92 | 1 (13.75)  0.78-0.93 |
|  |  | Day  0 | Day  1 | Day  30 | Day  60 | Day  90 | Day  120 | Day  150 | Day 180 | Day 210 | Day  240 | Day  270 | Day 300 | Day  330 | Day  360 |
|  |  | Deaths (%)  95% CI | | | | | | | | | | | | | |
| Fluralaner | 24h | 5 (6.25)  0.87-0.98 | 80 (100) | 73 (91.25)  0.02-0.14 | 39 (48.75)  0.40-0.62 | 30 (37.5)  0.51-0.73 | 19 (23.75)  0.66-0.85 | 0 (0)  0.99-1.00 | 0 (0)  0.99-1.00 | 0 (0)  0.99-1.00 | 0 (0)  0.99-1.00 | 0 (0)  0.99-1.00 | 0 (0)  0.99-1.00 | 0 (0)  0.99-1.00 | 0 (0)  0.99-1.00 |
|  | 48h | 3 (10)  0.82-0.96 | NA | 7 (100) | 24 (69)  0.12-0.30 | 18 (60)  0.29-0.50 | 19 (47.5)  0.41-0.63 | 7 (8.75)  0.85-0.97 | 1 (1.25)  0.96-1.00 | 1 (1.25)  0.96-1.00 | 0 (0)  0.99-1.00 | 2 (2.5)  0.94-1.00 | 0 (0)  0.99-1.00 | 4 (5)  0.90-0.99 | 0 (0)  0.99-1.00 |
|  | 72h | 0 (10)  0.82-0.96 | NA | NA | 11 (76)  0.01-0.13 | 8 (70)  0.19-0.40 | 9 (58.75)  0.30-0.52 | 15 (27.5)  0.62-0.82 | 8 (11.25)  0.81-0.95 | 7 (10)  0.83-0.96 | 2 (2.5)  0.94-1.00 | 3 (6.25)  0.88-0.99 | 2 (2.5)  0.94-1.00 | 1 (6.25)  0.88-0.99 | 3 (3.75)  0.92-1.00 |
|  | 96h | 0 (10)  0.82-0.96 | NA | NA | 6 (100) | 24 (100) | 12 (73.75)  0.16-0.35 | 29 (58.25)  0.25-0.46 | 23 (40)  0.49-0.70 | 20 (35)  0.54-0.75 | 16 (22.5)  0.68-0.86 | 12 (21.25)  0.69-0.87 | 7 (11.25)  0.81-0.95 | 5 (12.5)  0.80-0.94 | 7 (12.5)  0.80-0.94 |
|  | 120h | 3 (13.75)  0.77-0.93 | NA | NA | NA | NA | 21 (100) | 29 (100) | 26 (72.5)  0.17-0.37 | 22 (62.5)  0.26-0.48 | 31 (61.25)  0.28-0.49 | 23 (50)  0.39-0.60 | 25 (42.5)  0.46-0.68 | 9 (23.75)  0.66-0.85 | 2 (15)  0.77-0.92 |

Deaths: number of sand flies dead at the time observed.

%: cumulative percentage of deaths at each time observed.

95% CI: interval of the probability of survival at a specific time calculated using the Kaplan Meier method.

NA: samples showed 100% of death at the previous time.
